# Establishment of an efficient *in vitro* propagation system for *Iris sanguinea*

**DOI:** 10.1038/s41598-018-35281-y

**Published:** 2018-11-20

**Authors:** Ling Wang, Yu Du, Md. Mahbubur Rahman, Biao Tang, Li-Juan Fan, Aruna Kilaru

**Affiliations:** 1 The College of Landscape Architecture, Northeast Forestry University, Harbin, 150040 China; 2 Department of Biological Sciences, East Tennessee State University, Johnson City, TN 37614 USA

## Abstract

*Iris sanguinea* is a perennial flowering plant that is typically cultivated through seeds or bulbs. However, due to limitations in conventional propagation, an alternate regeneration system using seeds was developed. The protocol included optimization of sterilization, stratification and scarification methods as iris seeds exhibit physiological dormancy. In addition to chlorine-based disinfection, alkaline or heat treatment was used to break seed dormancy and reduce contamination. When seeds were soaked in water at 80 °C overnight, and sterilized with 75% EtOH for 30 s and 4% NaOCl solution for 20 minutes, contamination was reduced to 10% and a 73.3% germination was achieved. The germinated seedlings with 2-3 leaves and radicle were used as explants to induce adventitious buds. The optimal MS medium with 0.5 mg L^−1^ 6-benzylaminopurine, 0.2 mg L^−1^ NAA, and 1.0 mg L^−1^ kinetin resulted in 93.3% shoot induction and a proliferation coefficient of 5.30. Medium with 0.5 mg L^−1^ NAA achieved 96.4% rooting of the adventitious shoots. The survival rate was more than 90% after 30 days growth in the cultivated matrix. In conclusion, a successful regeneration system for propagation of *I. sanguinea* was developed using seeds, which could be utilized for large-scale propagation of irises of ecological and horticultural importance.

## Introduction

Blood iris or *Iris sanguinea* is a perennial herbaceous flowering plant of *Iridaceae*; it is a popular ornamental plant for its beautiful shape with unbranched stems, bright and colorful flowers and adaptability. Irises are widely cultivated in overwintering areas such as Inner Mongolia, Liaoning, Jilin, and Heilongjiang, mostly for landscaping^1–3^. Blood irises bear cold-tolerant flowers and are also an excellent choice for cut flower cultivation. Most plant parts of *I. sanguinea* cultivars also have medicinal value. Rhizomes and roots are often used for reducing inflammation and detoxification; triterpenes from seeds can regulate glucose uptake and thus beneficial to treat metabolic disorders such as diabetes^3–5^. Despite the significant features of *I. sanguinea*, its cultivation and utilization are not well established in China^1,3,6^. As such, wild resources for *I. sanguinea* have been greatly damaged, and their population is rapidly declining due to reclamation of wetlands and environmental destruction^7^. In order to protect *I. sanguinea* from extinction, and exploit its resources^1^, it is pertinent to develop methods for its rapid propagation.

Generally, propagation of *Iris* species is accomplished asexually by splitting bulbs or rhizomes, and sexually by seeds^2,3^. Although under natural conditions, ramet (clonal) or seed propagation is the preferred mode for multiplying *I. sanguinea*, their large scale production is limited by factors that control the propagation efficiency such as cross pollination, poor seed production, the long juvenile period, and germination success^8^. With regards to cultivation of *I. sanguinea*, previous studies have focused mostly on breeding, cross compatibility, seed biology and seedling development, and flowering and pollination^2,4,9^.

While *in vitro* micropropagation is an effective method for rapid generation of *Iris* seedlings, the efficiency, availability, and sterility of an explant can be constraint. For example, although callus was induced from flower organs, efficient regeneration of adventitious buds was not achieved^6,10–12^. There was some success when sterile shoot tip and leaf of *I. sanguinea* were used as explant materials where tufted buds were obtained^13^. Bud rhizome and roots of *I. sanguinea* were also used to obtain aseptic plantlets through rhizome pathway^11,12^. The availability of these various explants, stem tip, rhizome, and floral organs is, however, subject to seasonal time constraints as the above ground part of the plant withers in autumn. Thus, to accelerate the propagation of *I. sanguinea*, using germinated seedlings as explants would be convenient, as seeds are not limited by seasons and can be stored for long periods. Currently, a complete tissue culture system with seeds of *I. sanguinea* as a source for explant with efficient organogenesis has not been developed. In this study, we developed a method to successfully disinfect the seeds of *I. sanguinea*, obtained from freshly harvested capsules or long-term storage, stratify and scarify and germinate them to generate young seedlings that are used as explants.

For decades, it is known the *de novo* organogenesis from an explant is dependent on the ratio of plant hormones such as auxin to cytokinin with higher ratio directing the root formation while the lower ratio promotes shoot formation^14^. A 100% regeneration of shoots from root cuttings of arabidopsis was demonstrated when the cytokinin concentration was in excess to that of auxin^15^. Several studies utilized the genetic tools available for arabidopsis to unravel the underlying molecular mechanisms and that regulate phytohormone signaling^16–20^. In arabidopsis, shoot formation by cytokinins was achieved by the activation of cytokinin receptors and homeodomain regulators associated with cytokinin biosynthesis and negative regulation of root initiation by reducing the auxin efflux^16,19^. Nevertheless, tissue differentiation and *de novo* organogenesis of pluripotent cells are directed by different developmental pathways that are species/tissue-specific^20^ and likely dependent upon the timing of the stem cell development^17^. Thus, the efficiency of regeneration is dependent upon the type of explant and species in addition to the concentrations and the varying combinations of auxins and cytokinins in the growth medium^10,13,16,20^.

Based on various studies on *in vitro* propagation of irises^10–13,21^, we developed an optimized tissue culture method, utilizing a combination of synthetic auxin, α-naphthalene acetic acid (NAA), and cytokinins, kinetin (KT) or 6-benzylaminopurine (6-BAP). Together, the methods developed in this study are expected to serve as a basis for large-scale production of *I. sanguinea* seedlings, generation and improvement of new varieties, and maintenance of germplasm resources.

## Results

Development of *in vitro* propagation methods for *I. sanguinea* relied on using seeds as the initial source material for generation of young seedlings that were used as explants. To this extent, seeds from capsules harvested during varying seasons and long-term storage were used.

### Seed contamination and germination are affected by the harvest period of the capsules

**Figure 1:**
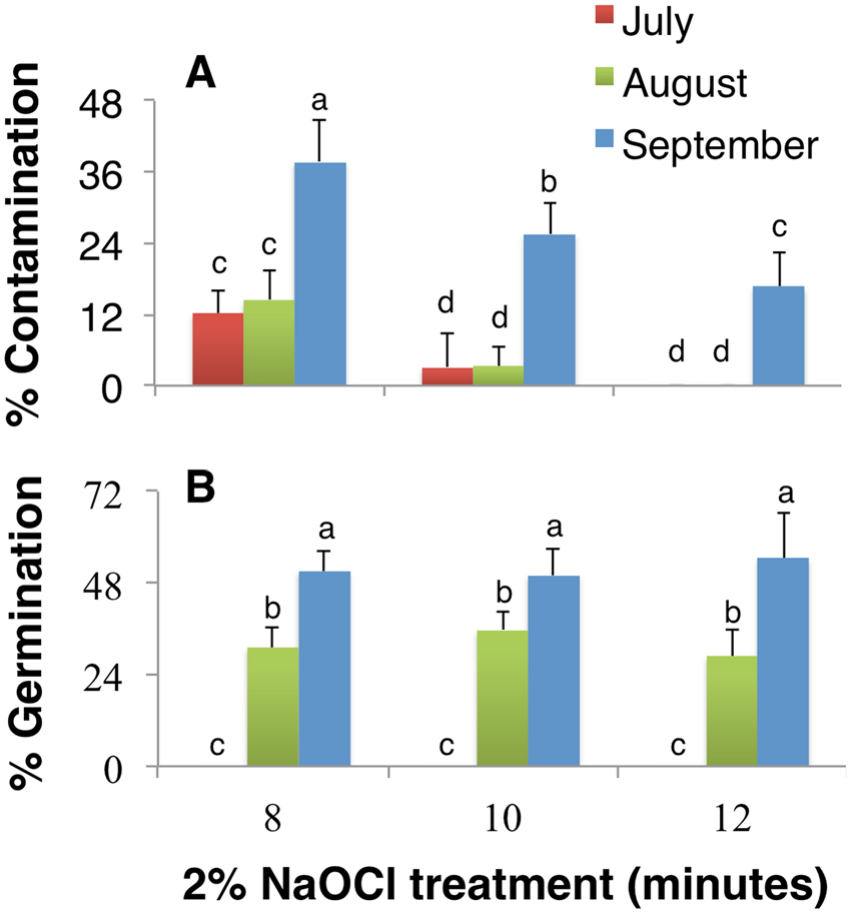
The effects of harvest period and disinfection methods on contamination and germination. The percentage of (**A**) contamination and (**B**) germination of *I. sanguinea* seeds obtained from capsules harvested in early July, August, and September after sterilized with 2% NaOCl for 8, 10, and 12 minutes. Values represent Mean ± SD of three biological replicates. Different letters on the bars indicate significant differences with each other (*P* < *0.05*), while same letters indicate the lack of significance, as determined by one-way analysis of variance (ANOVA) with Duncan’s post-test.

Seeds isolated from the capsules of *I. sanguinea*, harvested in the months of July, August, and September were subjected to disinfection with 75% EtOH for 30 s and 2% NaOCl for 8, 10, and 12 minutes (Table 1). Following disinfection, seed coats were removed with a scalpel and seeds were inoculated  on the germination media. In the subsequent days, contamination and germination were evaluated. Seeds inoculated from the capsules collected early in the season, July and August showed a low percentage of contamination when disinfected with 2% NaOCl for 8 or 10 minutes and no contamination was observed after 12 minutes of treatment (Fig. 1A). On the contrary, 16.7% contamination was observed for seeds from capsules collected in September even after 12 minutes of 2% sodium hypochlorite treatment. Irrespective of the harvest period of the capsules, the duration of sterilization with 2% NaOCl had a significant impact on contamination level (Fig. 1).

**Table 1:** The summary of pretreatment conditions for explant source materials.

Seed Source	Scarification/Statification		Disinfection	
	Treatment	Duration (h)	Treatment	Duration (min)
Capsules	None	None	2% NaOCl	8, 10, and 12
Seed from capusles	5% NaOH	2, 5, and 8	2% NaOCl	10
	10% NaOH	2, 5, and 8	2% NaOCl	10
Seeds from storage	Initial temperature of water set to 40, 70, 80, or 90 °C	Overnight	4% NaOCl	10, 20, and 30

Additionally, independent of the duration of hypochlorite disinfection, the different fruit picking periods significantly influenced seed germination. The later the harvest time, the higher was the percentage of germination (Fig. 1B). While the seeds from the capsules harvested in early July did not germinate, seeds from capsules harvested in August and September showed a moderate (30%) and the highest (54.4%) germination, respectively (Fig. 1B). Considering the high levels of contamination for seeds from September capsules, it can be deduced that harvesting capsules in early August and disinfection with 75% EtOH for 30 s followed by 2% NaOCl for 12 minutes would be an optimal choice for seed germination and explant generation.

### Alkaline scarification affects seed germination and mortality but not contamination

Iris seeds typically exhibit physiological dormancy and it is common to perform alkaline scarification, and/or stratification by soaking the seeds in cold or hot temperature to break the dormancy, prior to the removal of seed coat^8,22^.

**Figure 2:**
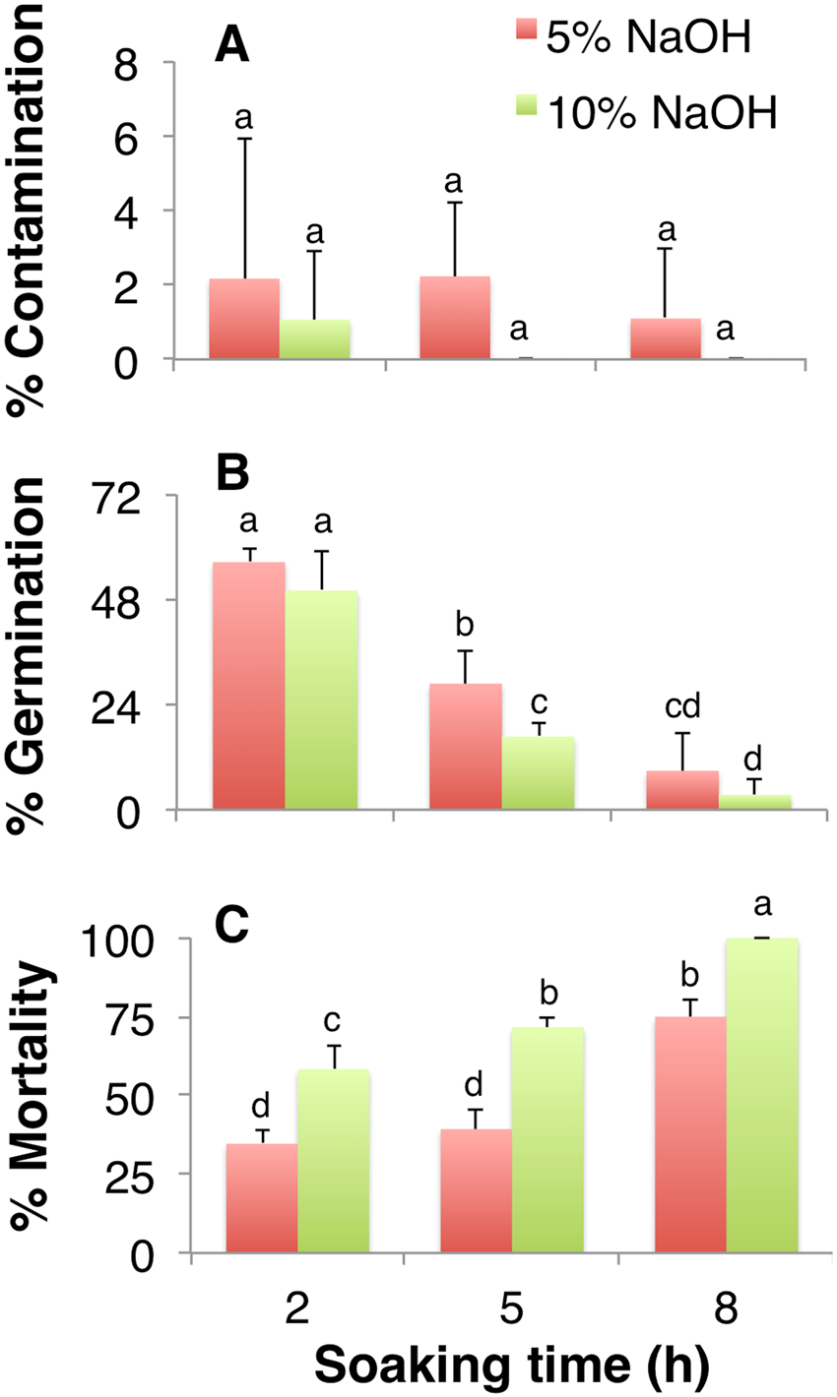
The effects of scarification, stratification and disinfection on stored seeds. The percentage of (**A**) contamination, (**B**) germination, and (**C**) mortality of seeds subsequent to seed coat removal after soaking in 5% or 10% NaOH solution for 2, 5 and 8 h. Values represent Mean ± SD of three biological replicates. Different letters on the bars indicate significant differences with each other (*P* < *0.05*), while same letters indicate the lack of significance, as determined by one-way analysis of variance (ANOVA) with Duncan’s post-test.

Here, the seeds of *I. sanguinea* underwent alkaline scarification with varying concentrations of NaOH (5% or 10%) and treatment duration (2, 5 or 8 h) prior to seed coat removal followed by hypochlorite disinfection (Table 1). The percentage of contamination in the inoculated seeds showed a declining trend with increase in NaOH concentration and the treatment duration. The difference in contamination between the treatments, however, was not significant; the highest percentage of contamination was only 2.2% (Fig. 2A).

The seed germination, however, declined by ~84% and ~94% when the treatment was prolonged from 2 to 8 h with 5% and 10% NaOH, respectively (Fig. 2B). More than 55% of seed germination, which was the highest, was achieved when the seeds were soaked for only two hours, independent of the NaOH concentration (Fig. 2B). One week after inoculation, some of the germinated seeds turned brown and eventually died; the percentage of mortality was affected by both concentration and duration of NaOH treatment (Fig. 2C). While the highest mortality was observed with prolonged soaking, the effect was significantly higher for seeds soaked in 10% NaOH than 5% (Fig. 2C). Even the 3.4% germination observed with seeds soaked for 8 h in 10% NaOH (Fig. 2B) subsequently browned and died at the later stage (Fig. 2C). Although the removal of seed coat for seeds treated with 5% NaOH for short duration (2 h) was difficult, soaking with 10% NaOH damaged the seeds and led to a significantly higher mortality. Thus, scarification of *I. sanguinea* seeds with 5% NaOH for 2 h was concluded to be optimal to achieve the highest percentage of germination (56.5%) with moderate contamination and least mortality (Fig. 2).

### Temperature stratification and hypochlorite treatment affects contamination and seed germination

**Figure 3:**
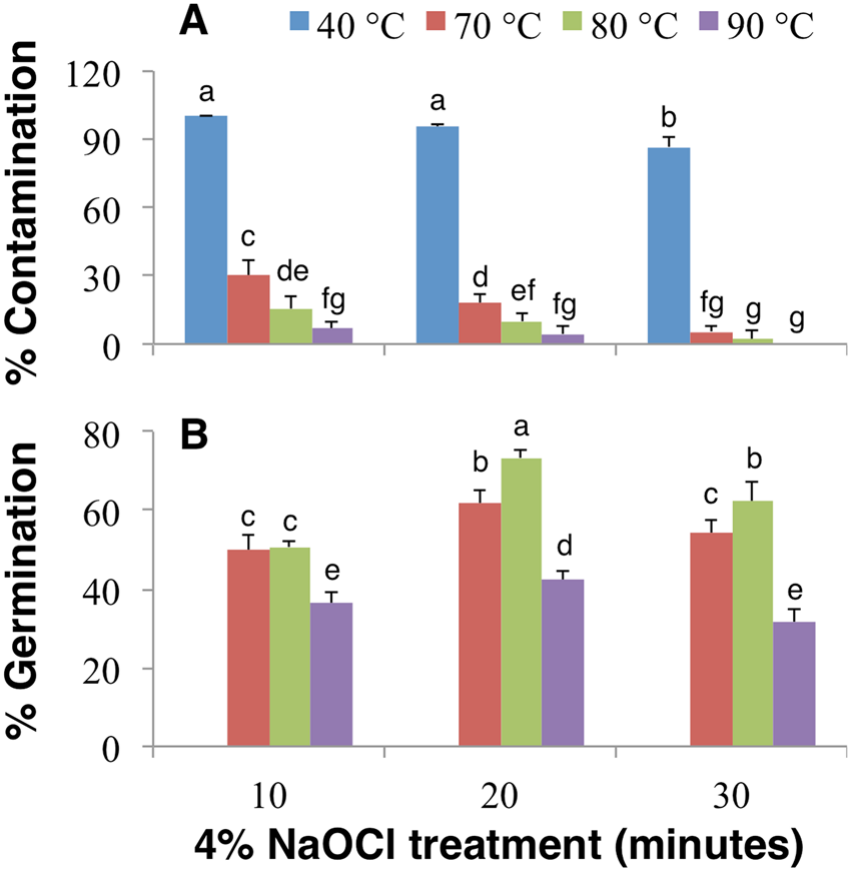
The effects of hot water and hypochlorite treatment on seeds. The percentage of (**A**) contamination and (**B**) germination of *I. sanguinea* seeds after soaking overnight in water at 40, 70, 80, or 90 °C and sterilization with 4% NaOCl for 10, 20, and 30 minutes. Values represent Mean ± SD of three biological replicates. Different letters on the bars indicate significant differences with each other (*P* < *0.05*), while same letters indicate the lack of significance, as determined by one-way analysis of variance (ANOVA) with Duncan’s post-test.

Hot water stratification is an effective method to induce dormancy break and also soften the seed coat prior to its removal. Therefore, *Iris* seeds were soaked in hot water at different temperatures overnight and subsequently disinfected with 4% NaOCl solution for 10 to 30 minutes (Table 1). Initial soaking temperature and the duration of disinfection treatment significantly affected both contamination and seed germination (Fig. 3). The seeds soaked in 40 °C warm water followed by 4% NaOCl disinfection showed the highest contamination; there was no germination even when the contamination was reduced to 85% with 30 minutes of disinfection (Fig. 3). Soaking the seeds in 70 °C water significantly reduced contamination, which was further reduced with prolonged hypochlorite treatment (Fig. 3A). Independent of the duration of NaOCl treatment, contamination was at its lowest when seeds were soaked in 80 °C and 90 °C water (Fig. 3A). Interestingly, germination was affected by both the duration of hypochlorite treatment and soaking temperature (Fig. 3B). The seeds soaked at 80 °C followed by 20 minutes of 4% NaOCl treatment showed the highest germination (73.3%), which was however, reduced significantly to 62% with 30 minutes of 4% NaOCl treatment (Fig. 3B). Higher temperature (90 °C), although effectively reduced contamination, negatively impacted seed germination, irrespective of the disinfection period (Fig. 3). Therefore, it is inferred that seeds soaked in water at 80 °C followed by 4% NaOCl disinfection for 20 minutes would achieve the highest percentage of germination, despite 10% contamination (Fig. 3).

### Cytokinins have a significant impact on the adventitious shoot proliferation of *I. sanguinea*

Previous studies with *I. sanguinea* have shown that while KT is necessary to induce organogenic calli or adventitious buds, direct organogenesis or shoot proliferation required 6-BAP in combination with auxin^21^. Thus, healthy germinated seedlings (2-3 leaves with radicle) of *I. sanguinea* were inoculated as explants on the culture media with varying concentrations and combinations of adenine derived cytokinins (6-BAP and KT) with synthetic auxin, NAA (Table 2). At 40 days after subculture, an optimal medium was selected based on the physical appearance of adventitious shoot growth, percentage of induction and multiplication or proliferation coefficient, which were significantly affected by the phytohormone concentration and the combination used (P < 0.05; Tables 2 and 3). The lowest concentrations of 6-BAP, NAA and KT (0.2, 0.2 and 0.5 mg L^−1^, respectively) showed an average response towards shoot induction and multiplication; the highest induction (93.3%) and multiplication rate (5.3) were achieved with a moderate increase in 6-BAP (0.5 mg L^−1^) and KT (1.0 mg L^−1^), but without altering the NAA content (Table 2). At the optimal 6-BAP concentration, however, the increase in NAA along with increase or decrease in KT content was not effective in promoting shoot proliferation. Interestingly, KT and 6-BAP together at their highest or lowest concentrations generated the lowest multiplication rate. The combination of the highest concentrations of KT and NAA also led to the least induction, with fragile and yellowish green shoots (53.3%; Table 2). The analysis of variance indicated that an optimal concentration of both 6-BAP and KT play a significant role in improving the adventitious shoot induction and proliferation coefficient (P < 0.01), while NAA only affected induction (P < 0.05) and not the multiplication coefficient (P > 0.05; Table 3).

**Table 2:** Orthogonal-array design for phytohormone treatments and their effect on adventitious shoot induction and proliferation.

Treatment^#^	Orthogonal array^a^			Phytohormone (mg L^−1^)			Induction^b^ (%)	Multiplication coefficient^b^	Adventitious shoot appearance
	A	B	C	6-BAP	NAA	KT			
1	1	1	1	0.2	0.2	0.5	68.3 ± 2.9 de	2.8 ± 0.3 e	*healthy and strong, bottle green*
2	1	2	2	0.2	0.4	1.0	81.7 ± 2.9 bc	3.8 ± 0.4 c	*healthy and strong, green*
3	1	3	3	0.2	0.6	2.0	53.3 ± 7.6 f	3.5 ± 0.2 cd	*thin and delicate, yellow green*
4	2	1	2	0.5	0.2	1.0	93.3 ± 2.9 a	5.3 ± 0.3 a	*healthy and strong, bottle green*
5	2	2	3	0.5	0.4	2.0	80.0 ± 5.0 bc	4.7 ± 0.3 b	*thin and delicate, yellow green*
6	2	3	1	0.5	0.6	0.5	71.7 ± 2.9 cd	3.0 ± 0.2 e	*healthy and strong, green*
7	3	1	3	1.0	0.2	2.0	61.7 ± 7.6 ef	2.9 ± 0.3 e	*thin and delicate, yellow green*
8	3	2	1	1.0	0.4	0.5	73.3 ± 7.6 bcd	3.2 ± 0.3 de	*healthy and strong, green*
9	3	3	2	1.0	0.6	1.0	83.3 ± 5.8 b	4.4 ± 0.4 b	*healthy and strong, yellow green*

^a^Numbers represent the levels in orthogonal array of 3 × 3 design; ^b^values are average and SD of three independent experiments and different letters indicate significant differences (P < 0.05), as determined by one-way analysis of variance (ANOVA) with Duncan's post-test.

**Table 3:** One-way analysis of variance of phytohormone influence on (A) % induction and (B) multiplication coefficient of adventitious shoot formation.

Factor	Sum of squares	df	Mean square	F	P – value
***A. Effect on % induction***					
NAA	357.407	2	178.704	5.259	0.015
6-BAP	890.741	2	445.370	13.106	0.000
KT	2124.074	2	1062.037	31.253	0.000
***B. Effect on multiplication coefficient***					
NAA	0.356	2	0.178	0.753	0.484
6-BAP	4.447	2	2.224	9.394	0.001
KT	10.287	2	5.144	21.731	0.000

### Exogenous NAA induces rooting of adventitious shoots

**Figure 4:**
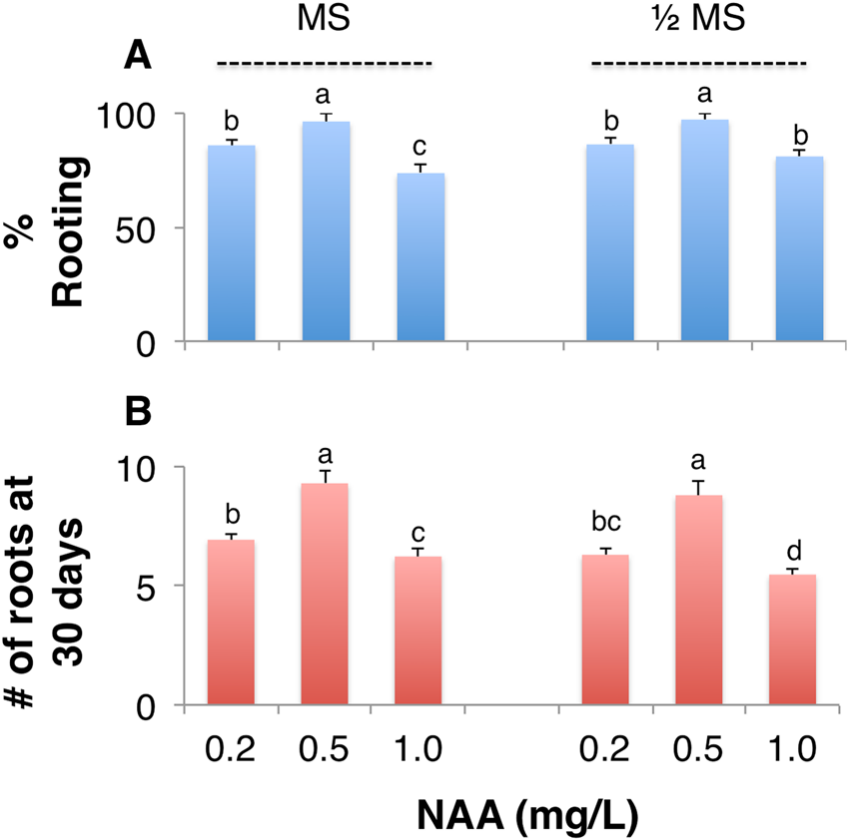
The effects of NAA and MS media on rooting of adventitious shoots. The percentage of (**A**) rooting, and (**B**) the number of roots formed at 30 days when adventitious shoots were cultured on MS or 1/2 MS media containing 0.2, 0.5, or 1.0 mg L^−1^ of NAA. Values represent Mean ± SD of three biological replicates. Different letters on the bars indicate significant differences with each other (*P* < *0.05*), while same letters indicate the lack of significance, as determined by one-way analysis of variance (ANOVA) with Duncan’s post-test.

After multiple subcultures, 5–10 cm long, strong, and well-grown adventitious shoots were selected, their leaves were cut at about 2 cm from the base and then inoculated into the rooting medium. Three concentrations of NAA on 1/2 or full MS medium were tested to determine the optimal rooting medium. Emergence of roots occurred after five days of inoculation in the rooting medium and roots were clearly visible from the bottom of the medium after seven days. After 30 days, roots grew to ~3 cm at which time their growth status was assessed (Table 4) and the percentage and the number of roots formed were quantified (Fig. 4). The percentage of rooting and the number of roots increased first and then decreased with the increase of NAA concentration in MS and 1/2 MS medium. There was no significant difference in the percentage of rooting and the number of roots formed between MS and 1/2 MS medium, when the concentration of NAA was 0.5 mg L^−1^; rooting growth was however, inhibited when the NAA was higher than 0.5 mg L^−1^ (Fig. 4). In later stages of growth, the adventitious shoots in 1/2 MS medium were not as healthy as those in the full-strength MS medium and appeared to turn greenish yellow (Table 4). The optimal rooting medium for adventitious shoots was thus determined to be MS medium with 0.5 mg L^−1^ NAA.

**Table 4:** Growth status of root from adventitious shoots on various rooting media.

Medium	NAA (mg L^−1^)	Root appearance	Seedling appearance
MS	0.2	*Slender and relatively dense*	*Strong and dark green*
MS	0.5	*Thick and dense*	*Strong and dark green*
MS	1.0	*Short, thick and partial malformation*	*Weak and greenish yellow*
1/2MS	0.2	*Slender and relatively dense*	*Greenish yellow*
1/2MS	0.5	*Thick and dense*	*Greenish yellow*
1/2MS	1.0	*Short, thick and partial malformation*	*Weak and greenish yellow*

### Tissue culture plantlets of *I. sanguinea* demonstrated high survival in greenhouse

**Figure 5:**
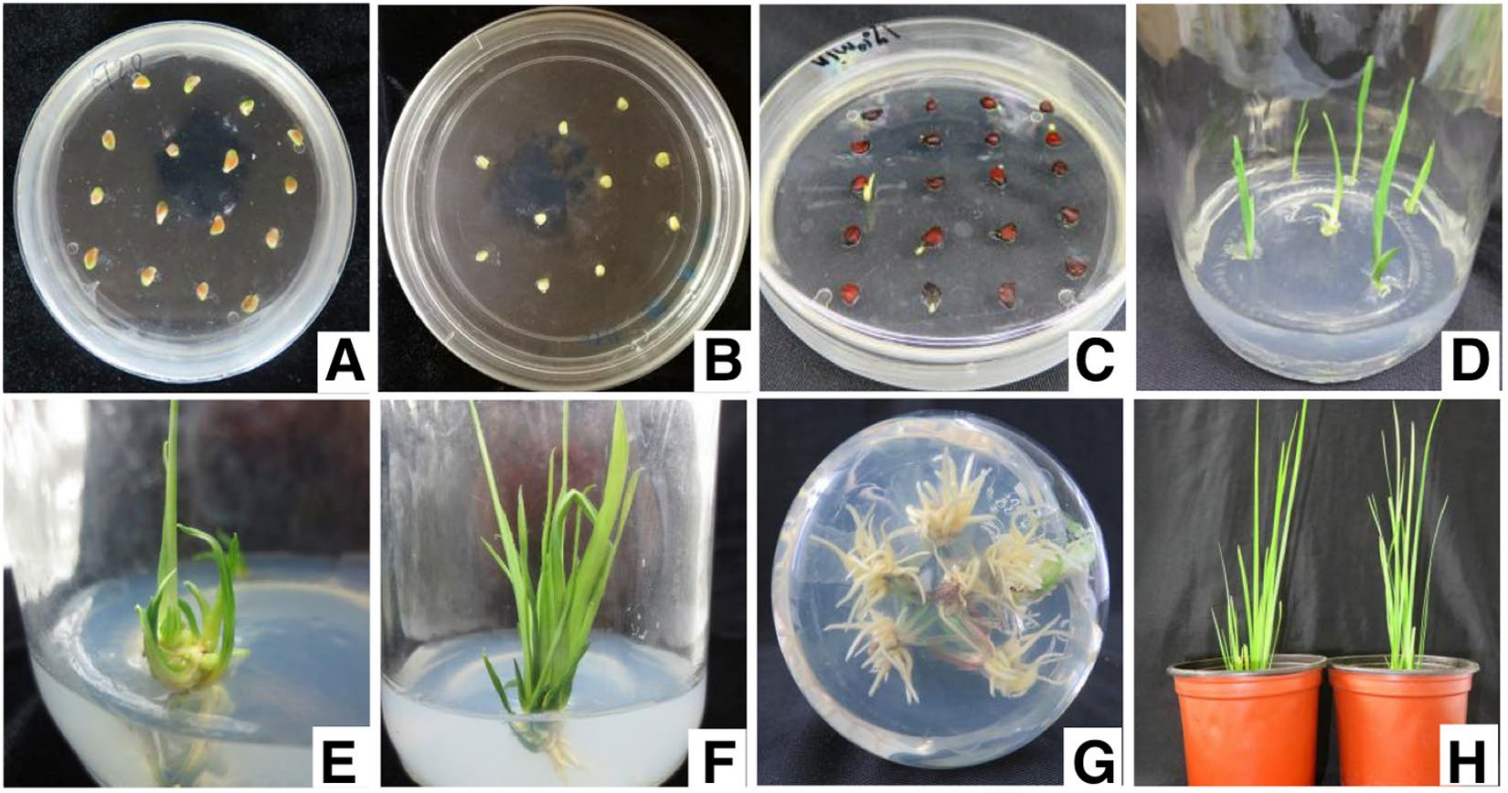
Representative images of stages in tissue culture from seeds to plants. Inoculated seeds of *I. sanguinea* from (**A**) sterile indehiscent capsule and soaked in NaOH, or (**B**) hot water; (**C**) germinated seeds and (**D**) seedlings; (**E**) induced adventitious shoots at 20 and (**F**) 40 days; (**G**) clustered shoots with induced roots (noticeable from the bottom of the jar); (**H**) transplanted plantlets established after 30 days.

The process of adventitious shoot induction and regeneration began with germination of disinfected seeds, which occurred after ~10 days of inoculation (Fig. 5A–C), followed by the selection of seedlings (2–3 leaves with radicle) and their inoculation in the induction medium for adventitious shoot formation and proliferation (Fig. 5D). After cultured for 40 days, an average of five shoots were differentiated from each initially inoculated adventitious bud (Fig. 5E,F). Subsequent to rooting and stable growth of clumped, regenerated shoots (Fig. 5G), robust plantlets were separated and transplanted into the cultivation medium. After 30 days of transplantation and hardening, healthy growth with 93.3% survival was achieved in the greenhouse (Fig. 5H).

## Discussion and Conclusions

Although tissue culture propagation of *Irises* has been accomplished with various explants, continuous availability of the source of explant and its aseptic nature, and ability to respond to induction and propagation methods are, however, crucial for the successful establishment of a regeneration protocol^6,10,12^. Disinfection of the source material prior to inoculation or isolation of explant is essential as microorganisms that reside on the explant surface may find the *in vitro* environment optimal for their growth and affect the overall success of tissue culture^6,10,23^. Direct contact of the explants with disinfectants, in order to fully eliminate the contaminants, can severely affect their regeneration potency^10^. Concentration and application period of the disinfectants can also drastically reduce the regeneration capacity and viability of the explant. Sterilization with 75% EtOH treatment increases permeability, and prolonged soaking can result in decolorization, injury and toxicity, as in *Iris ensata*^8,24^. Depending on the likely microbial contaminants, several surface disinfectants such as ethanol, NaOCl, H_2_O_2,_ AgNO_3,_ HgCl_2_, and bromine water are commonly used for surface sterilization, prior to *in vitro* culture^23,25,26^.

In tissue culture of various *Irises*, NaOCl was used as a disinfectant as it is highly effective against bacteria, fungi, and viruses^10,11,26,27^. High contamination in seeds from capsules harvested in early September (Fig. 1) is likely due to the dehiscent nature of the capsule at maturity; seeds in such capsules are likely to be exposed to contaminants and are poorly disinfected. On the contrary, seeds from indehiscent and unripe capsules from early August showed little contamination after 10 minutes of disinfection but germination was moderate at about 36% (Fig. 1). Seeds from long-term storage on the other hand, subsequent to alkaline scarification followed by hypochlorite treatment, resulted in about ~59% germination. These data together suggest that seeds from long-term storage, relative to those from freshly harvested capsules will serve as an efficient source of explant generation.

Seed coat in irises and several other plants plays an inhibitory role during germination as it retains dormancy due to inhibitors in the seed coat, and by preventing water penetration, gas exchange, and acting as a mechanical barrier for the embryo^8,28^. Therefore, various methods for scarification, stratification, and seed coat removal have been developed to break the seed dormancy and improve germination^8,10,22,29–31^. Specifically, alkaline (NaOH) scarification is known to increase the permeability of the seed coat, leading up to dormancy break^22,29^. In most *Iris* species, germination was significantly improved with the removal of seed coat, which results in increased membrane permeability, and removes some endogenous inhibitors^8,28^. In blood iris seeds, a 93.3% germination was achieved when the endosperm was resected from the micropylar end, while the removal of seed coat alone accomplished 55.6% germination^8^. About 3.3% germination observed without the removal of seed coat suggests its strong inhibitory role in germination^8^. In this study, removal of seed coat for seeds of *I. saguinea*, after soaking them in 5% NaOH for 2 h showed about 56.5% germination while prolonged treatment likely damaged the embryo (Fig. 2).

Soaking seeds in hot or cold water for the purpose of stratification is often used in addition to chemical scarification^29,31^. In this study, seeds soaked in water with initial temperature of 80 °C overnight resulted in the highest germination (~73%) compared to 5% NaOH treatment for 2 h (~56.5%) (Figs 2 and 3). Hot water soaking allowed for efficient removal of seed coat and also was effective in reducing contamination and thus improved germination^8^. In summary, while the tested methods of scarification and stratification improved germination and reduced contamination, hot water treatment would be the preferred choice (Figs 2 and 3); if the removal of endosperm would further enhance seed germination remains to be tested.

Plant growth regulators play important roles in regulating cell differentiation and organogenesis. It is well established that the growth media containing cytokinin promotes shoot formation in micropropagation and a higher ratio of cytokinin to auxin is required for shoot regeneration^14,16,17,19^. Studies with various *Iris* species have also shown that a complete plantlet can be directly induced from scape, stem tip, rhizome, and seed embryo by using a wide range of cytokinins including KT, 6-BAP, and ZT^10–12,24,32^. Previously, using shoot tip as an explant and with only 6-BAP (1.0 mg L^−1^) and NAA (0.2 mg L^−1^) in the media, a 70% shoot induction was achieved but with a low 2.75 multiplication coefficient. Among the cytokinins, KT was shown to be effective in generating embryogenic calli and increasing the proliferation rate in explants of various *Iris* species, including *I. sanguinea*^10,21^. Here, when the concentration of 6-BAP was maintained constant, KT at a lower concentration than the optimal (1.0 mg L^−1^), resulted in less proliferation of shoots (Table 2). By reducing the 6-BAP concentration (0.5 mg L^−1^) and adding KT at 1.0 mg L^−1^ to the media a 93.3% shoot induction with 5.34 proliferation coefficient was achieved (Table 2), suggesting a significant role for KT in increasing the proliferation rate. While higher concentration of cytokinins can negatively affect shoot formation and regeneration, it is well established that a synergistic effect of different cytokinins can result in effective regeneration rates^16,19^.

Although a higher ratio of auxin to cytokinin induces root formation^20^, like with cytokinins, increased concentration beyond the optimal level for root formation often results in inhibition, as with the regenerated seedlings of arabidopsis^33^. In this study, more than 96.4% rooting with 9.3 roots/plant was observed 30 days after inoculation into rooting medium MS and NAA at 0.5 mg L^−1^ (Table 4), with 93.3% survival of plantlets when transferred to potting soil.

One of the main goals of this study was to successfully develop a regeneration protocol that does not rely on limited source material for explants such as stem tip or rhizome of *I. sanguinea*, which may be limited by seasons. By establishing the regeneration of adventitious shoot formation of *I. sanguinea* with seeds from long-term storage as the main source resolves the problem of generating explant material that is limited by seasons. Furthermore, we demonstrated that stored seeds soaked in 80 °C water overnight followed by 4% NaOCl disinfection for 20 minutes result in successful germination. Subsequently, efficient adventitious shoot induction and proliferation was accomplished by culturing the germinated seedlings in the media with 0.5 mg L^−1^ 6-BAP, 1.0 mg L^−1^ KT, and 0.2 mg L^−1^ NAA. Transferring the regenerated shoots into rooting media with NAA in MS resulted in healthy plantlets that were successfully established in the greenhouse. These results could be extended to other *Iris* species as well for their successful commercial/large-scale propagation.

## Materials and Methods

### Plant materials

Seeds of *I. sanguinea* were collected from Mao’er Mountain Experimental Station, Northeast Forestry University, Harbin, China, in September 2015 and kept in a dry place at room temperature. Seed capsules were collected from the Nursery of the College of Landscape Architecture, Northeast Forestry University, Harbin, China from July to September in 2016.

### Experimental method

The experiment was carried out to establish seeds as an aseptic source material for explant generation. Disinfection methods for seeds obtained from freshly harvested capsules in varying months were tested by the quantification of contamination and germination of inoculated seeds. Additionally, scarification and/or stratification and disinfection methods were also tested for previously harvested and stored seeds. The adventitious shoot regeneration and proliferation was optimized with varying concentrations of growth regulators 6-BAP, NAA and KT. Subsequently, rooting and transplanting conditions were also established. All experiments were conducted in a tissue culture chamber at 25 ± 1 °C with a light intensity of 25 μmol m^−2^ s^−1^ for 14 h d^−1^.

### Disinfection of capsules and its seeds

Fruit capsules from multiple plants were collected and placed in clean sulphuric acid paper bags for good air permeability. The capsules were rinsed with detergent for 5–10 minutes and washed with tap water to remove surface impurities. Washed capsules were dried on the gauze at room temperature to prevent subsequent dilution of disinfectant from the surface water. In a laminar flow cabinet, the dried capsules were placed in a tissue culture bottle and sterilized with 75% EtOH for 30 s with shaking. After that, capsules were washed with sterile water for three times, and were treated with 2% NaOCl solution for 8, 10 or 12 minutes (Table 1) and then washed again for 3–5 times with sterile water. Seeds were isolated from the disinfected capsule and the seed coats were removed using a sterilized scalpel. Naked seeds were used for direct inoculation on germination medium.

### Scarification, Stratification, and disinfection of stored seeds

Stored seeds were first stirred and cleaned with diluted detergent water. After removing the impurities, seeds were placed under running water for 5–10 minutes until no foam was produced. Sterile gauze was used to absorb excess water from the surface of the seeds. A portion of these stored seeds was treated with either 5% or 10% NaOH solution in a tissue culture bottle at room temperature for 2, 5 or 8 h (Table 1). The NaOH treated seeds were then placed into a nylon net bag and seed coat was removed by rubbing the bag. These naked seeds were rinsed under running water for 30 minutes in a tissue culture bottle and were further disinfected with 2% NaOCl solution for 10 minutes (Table 1) and dried at room temperature.

Another portion of the stored seeds was divided into four groups and incubated separately in a water bath overnight with initial temperatures set at 40, 70, 80, and 90 **°**C; the following day seeds were dried at room temperature. Seed coat was removed for these stratified seeds and were further disinfected with 4% NaOCl solution for 10, 20 and 30 minutes (Table 1). After disinfection, naked seeds were washed and dried on a sterile filter paper, and prepared for inoculation on germination medium.

### Media preparation and inoculation

Standard MS (Murashige and Skoog)^34^ media was prepared by adding 4.74 g L^−1^ MS powder and 30 g L^−1^ sucrose with pH adjusted to 5.8; no additional buffer was used to maintain the pH. Media was sterilized by autoclaving after adding 3 g L^−1^ agar. All Petri dishes (90 mm) and forceps were also sterilized by autoclaving and were further disinfected under ultraviolet light for 20 minutes in a laminar cabinet. About 30 mL MS media was poured into Petri dish and left to solidify. Thirty seeds with seed coats removed were evenly distributed onto the surface of the MS media for germination. Petri dishes were prepared in three replicates. Inoculated plates were sealed with parafilm and placed in a tissue culture room at 25 ± 1 °C with a light intensity of 25 μmol m^−2^ s^−1^ for 14 h d^−1^. Seed contamination was evaluated on the 15^th^ day and germinated seeds were recorded on the 30^th^ day.

### Isolation of explant and shoot induction

Healthy germinated seedlings with two to three leaves and radicle were inoculated on the adventitious shoot induction media. The basal MS medium was supplemented with 6.0 g L^−1^ agar, 30 g L^−1^ of sucrose and varying concentrations and combinations of growth regulators, 6-BAP, NAA and KT (Table 2); pH was adjusted to 5.8. A wide-mouth tissue culture bottle (330 mL) with 40 to 60 mL of culture medium was used for each treatment. The culture bottle was capped and sterilized by autoclaving for 20 minutes and left for 3 to 4 days to allow for evaporation of condensed water. This duration also allowed for detecting and removal of jars with any microbial contamination that may have resulted from improper handling during media preparation. An optimal induction medium was determined upon evaluation of adventitious shoot formation and proliferation after 40 days; this medium was also used for further subculture of the adventitious shoots.

### Rooting culture

Vigorously growing clusters with 3 to 4 adventitious shoots were separated or cut with minimal damage and inoculated as single units for rooting. Basal or 1/2 MS media with 0.2, 0.5, or 1.0 mg L^−1^ NAA were used to determine the optimal rooting medium. Each treatment was conducted in triplicates by inoculating five clusters of ~20 shoots. The number of roots formed was counted at the 30^th^ day of root induction.

### Transplantation of rooted plantlets

Rooting jars, with uniform and healthy shoot growth were selected, and the caps were left open after adding some aseptic water to the jar. The plantlets were allowed to adjust to the new growth conditions for two days and were carefully removed from the jar and rinsed with sterile water to eliminate media remnants. Clean plantlets were transplanted into pots containing orchard soil and vermiculite in a ratio of 2:1 for further development of the plant in the greenhouse at 25 ± 1 °C with a light intensity of 25 μmol m^−2^ s^−1^ for 14 h d^−1^. The survival of transplanting was determined after 30 days.

### Data analyses

The following formulae were used for calculating each of the parameters reported in the study:

$$\begin{array}{c} \% Contamination=(\#of\,contaminated\,seeds/\#\,of\,inoculated\,seeds)\times 100\\  \% Germination=(\#of\,germinated\,seeds/\#of\,inoculated\,seeds)\times 100\\  \% Mortality=(\#of\,brown\,or\,dead\,seedlings/\#of\,inoculated\,seeds)\times 100\\  \% Induction=(\#of\,induced\,adventitious\,shoots/\#of\,inoculations)\times 100\\  \% Rooting=(\#of\,adventitious\,shoot\,with\,rooting/\#of\,inoculated\,shoots)\times 100\\ Multiplication\,coefficient=\#of\,shoots\,after\,multiplication/\#of\,inoculated\,seedlings.\end{array}$$

The data were processed and results were analyzed using a previously described method by SPSS2.0 software^35^. Data were expressed as their mean value and standard deviation (SD). One-way analysis of variance (ANOVA) was used to determine the significance of the various effects studied. Duncan’s Multiple Range Test was used for pair-wise comparison of the data.
